# Automated anatomical labeling of the intracranial arteries via deep learning in computed tomography angiography

**DOI:** 10.3389/fphys.2023.1310357

**Published:** 2024-01-04

**Authors:** Ting Chen, Wei You, Liyuan Zhang, Wanxing Ye, Junqiang Feng, Jing Lu, Jian Lv, Yudi Tang, Dachao Wei, Siming Gui, Jia Jiang, Ziyao Wang, Yanwen Wang, Qi Zhao, Yifan Zhang, Junda Qu, Chunlin Li, Yuhua Jiang, Xu Zhang, Youxiang Li, Sheng Guan

**Affiliations:** ^1^ Department of Interventional Neuroradiology, Beijing Tiantan Hospital and Beijing Neurosurgical Institute, Capital Medical University, Beijing, China; ^2^ School of Biomedical Engineering, Capital Medical University, Beijing, China; ^3^ Department of Neurointerventional Engineering and Technology, Beijing Engineering Research Center (NO: BG0287), Beijing, China; ^4^ China National Clinical Research Center for Neurological Diseases, Beijing, China; ^5^ Department of Neurosurgery, Beijing Chaoyang Hospital, Capital Medical University, Beijing, China; ^6^ Department of Radiology, Third Medical Center of Chinese PLA General Hospital, Beijing, China; ^7^ Department of Interventional Neuroradiology, The First Affiliated Hospital of Zhengzhou University, Zhengzhou, Henan, China; ^8^ Key Laboratory of Fundamental Research on Biomechanics in Clinical Application, Capital Medical University, Beijing, China

**Keywords:** computed tomography angiography, intracranial arteries, deep learning, anatomical labeling, intracranial aneurysm, arterial stenosis

## Abstract

**Background and purpose:** Anatomical labeling of the cerebral vasculature is a crucial topic in determining the morphological nature and characterizing the vital variations of vessels, yet precise labeling of the intracranial arteries is time-consuming and challenging, given anatomical structural variability and surging imaging data. We present a U-Net-based deep learning (DL) model to automatically label detailed anatomical segments in computed tomography angiography (CTA) for the first time. The trained DL algorithm was further tested on a clinically relevant set for the localization of intracranial aneurysms (IAs).

**Methods:** 457 examinations with varying degrees of arterial stenosis were used to train, validate, and test the model, aiming to automatically label 42 segments of the intracranial arteries [e.g., 7 segments of the internal carotid artery (ICA)]. Evaluation metrics included Dice similarity coefficient (DSC), mean surface distance (MSD), and Hausdorff distance (HD). Additionally, 96 examinations containing at least one IA were enrolled to assess the model’s potential in enhancing clinicians’ precision in IA localization. A total of 5 clinicians with different experience levels participated as readers in the clinical experiment and identified the precise location of IA without and with algorithm assistance, where there was a washout period of 14 days between two interpretations. The diagnostic accuracy, time, and mean interrater agreement (Fleiss’ Kappa) were calculated to assess the differences in clinical performance of clinicians.

**Results:** The proposed model exhibited notable labeling performance on 42 segments that included 7 anatomical segments of ICA, with the mean DSC of 0.88, MSD of 0.82 mm and HD of 6.59 mm. Furthermore, the model demonstrated superior labeling performance in healthy subjects compared to patients with stenosis (DSC: 0.91 vs. 0.89, *p* < 0.05; HD: 4.75 vs. 6.19, *p* < 0.05). Concurrently, clinicians with model predictions achieved significant improvements when interpreting the precise location of IA. The clinicians’ mean accuracy increased by 0.04 (*p* = 0.003), mean time to diagnosis reduced by 9.76 s (*p* < 0.001), and mean interrater agreement (Fleiss’ Kappa) increased by 0.07 (*p* = 0.029).

**Conclusion:** Our model stands proficient for labeling intracranial arteries using the largest CTA dataset. Crucially, it demonstrates clinical utility, helping prioritize the patients with high risks and ease clinical workload.

## 1 Introduction

Cerebrovascular diseases, such as aneurysms, stenosis, and arteriovenous malformations, are leading causes of death and disability (GBD, 2015 Mortality and Causes of Death Collaborators, 2016). The intrinsic characteristics of intracranial arteries enables to aid in understanding the disease pathogenesis that causes the morphology change and dysfunction of specific arterial segment and manifests as related clinical symptoms (Turan et al., 2010; Mackey et al., 2012). Hence, precise anatomical labeling of the intracranial arteries is crucial for physicians to understanding the mechanism, diagnosis, and treatment of cerebrovascular conditions. Although consensus on defining the fine segments of intracranial arteries through imaging and anatomy has been established (Bouthillier, van Loveren, and Keller, 1996; Yavagal and Haussen, 2011; Harrigan and Deveikis, 2018), manual labeling of fine segments of intracranial arteries is time-consuming and prone to inter-and intra-observer variability. Furthermore, this problem is exacerbated due to the lack of experienced radiologists given the increasing imaging data. Previous studies on anatomical labeling of intracranial arteries have been constrained by limited datasets and only performed on magnetic resonance angiography (MRA) images (Dunas et al., 2016; Robben et al., 2016; Dunas et al., 2017). Additionally, computed tomography angiography (CTA) seems to be more encouraging in the assessment of aneurysms, vessel stenosis and patency (Koelemay et al., 2004; Manniesing et al., 2008). Consequently, automated anatomical labeling of cerebral vasculature with detailed segments on CTA is urgent and essential for the diagnosis and treatment schemes of arterial diseases.

Deep learning (DL) has shown significant potential in medical image analysis tasks. Notably, the U-Net framework with symmetric network architecture is widely adopted in the field of medical image segmentation because of its flexibility and achieves remarkable successes (Jin et al., 2020; Klimont et al., 2020; Mubashar et al., 2022). Previous models have focused on the segmentation of the specific artery (e.g., the carotid artery) or the whole 3D cerebral vessels (Groves et al., 2020; Klimont et al., 2020; Bortsova et al., 2021; Guo et al., 2021). Only few studies strive for the automatic segmentation or labeling of intracranial arteries involved with detailed segments (i.e., automatic labelling of fine segments).

So far, this is the first study to develop a powerful U-Net-based DL model for labeling the most detailed segments of intracranial arteries on CTA scans. Additionally, 7 segments of the internal carotid artery (ICA) were successfully labeled for the first time where stenosis and aneurysms frequently occur. Moreover, the trained DL algorithm was applied in a clinical experiment to assess the impact on the precise intracranial aneurysm (IA) localization. The performance of 5 expert raters with different experience levels as to localization accuracy, clinical decision time, and the interrater agreement was analyzed without and with the support of artery classification by the DL algorithm, for a washout period of 14 days between two interpretations.

## 2 Materials and methods

### 2.1 Dataset

**FIGURE 1:**
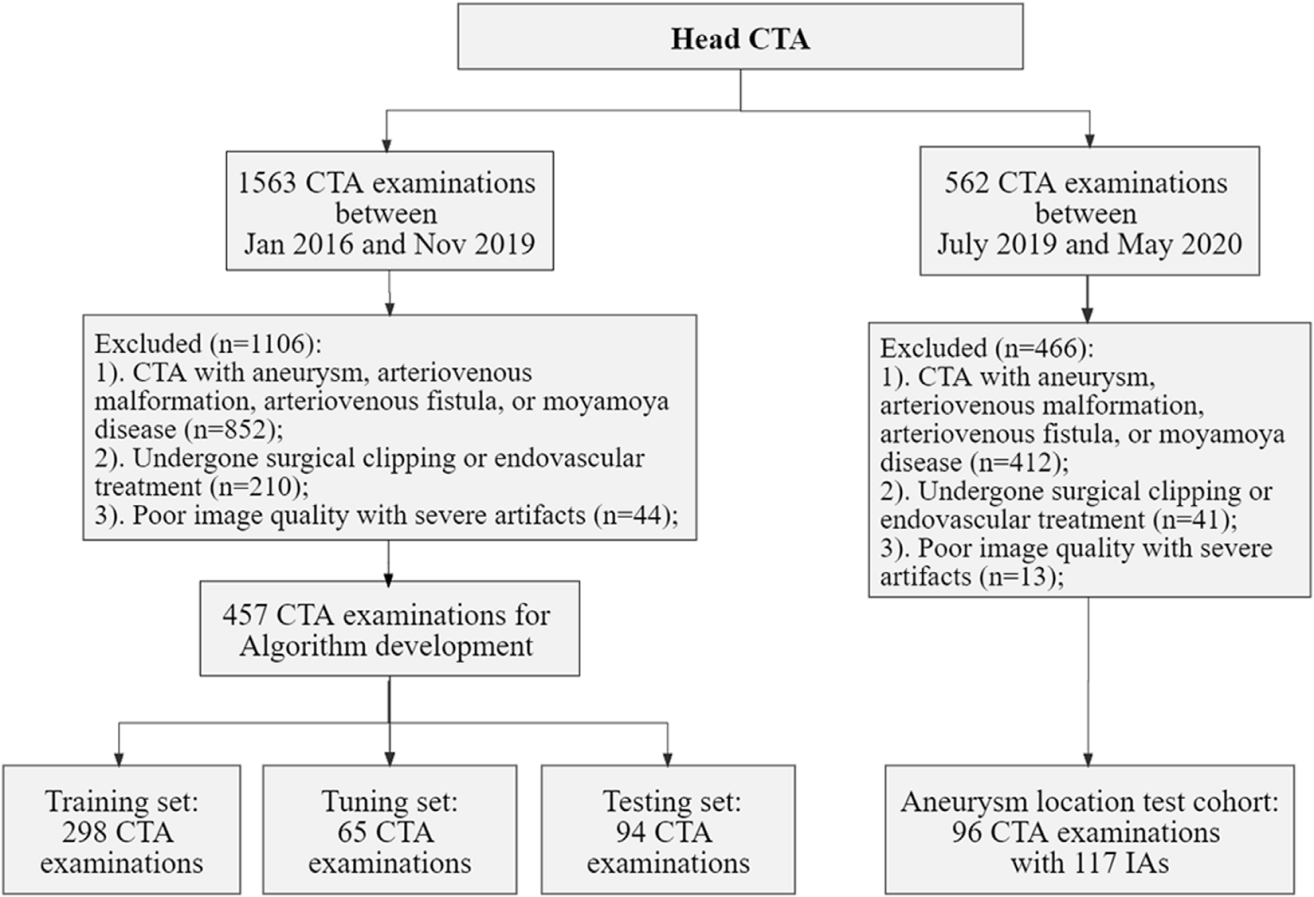
Flowchart of data acquisition, selection, and assignment. CTA, computed tomography angiography; IAs, intracranial aneurysms.

The local Institutional Review Board (IRB) approved this retrospective study, waiving the requirement for informed consent, in adherence to the principles of the Declaration of Helsinki. Data for model development were selected from head CTA images, with or without arterial stenosis, in the imaging database of our hospital between January 2016 and November 2019. The exclusion criteria included patients with intracranial aneurysms (IAs), arteriovenous malformation, arteriovenous fistula, Moyamoya disease, and poor image quality. Dataset for validation of the proposed model in real-world clinical scenarios, the inclusion criteria included head CTA images with at least one IA and without other arterial diseases, with digital subtraction angiography (DSA) verification from July 2019 to May 2020 in the same hospital. The flowchart of data acquisition, selection, and assignment were depicted in Figure 1.

Collectively, we included a total of 457 CTA examinations performed on GE Healthcare scanners for model development, which were randomly divided into training (*n* = 298), tuning (*n* = 65), and testing sets (*n* = 94). Furthermore, 96 CTA examinations with 117 aneurysms were included for the validation. The severity of artery stenosis was graded as follows (Zhang et al., 2015; Fu et al., 2023): mild stenosis (<50%), moderate stenosis (50%–70%), severe stenosis (>70%) and occlusive (100%). In case of multiple stenosis, the most severe stenosis was adopted.

### 2.2 CTA image acquisition, reconstruction and preprocessing

Standard head CT angiography examinations were acquired on axial section with post-processing reconstruction on sagittal, coronal, maximum intensity projection (MIP) and 3-dimensional volume rendered (3D-VR) views as necessary. All included CTA examinations were acquired on axial section with Discovery CT750 HD (GE Healthcare, Chicago, IL, United States) utilizing a slice thickness of 0.63 mm, a tube voltage of 100 kVp, and the effective tube current ranging between 2 and 3 mAs. It is worth noting that DSA images are auxiliary data serving as a reference for enrollment of IAs and are not fed into the proposed model.

For image preprocessing, normalization, spatial resampling, and extraction of binary vessel masks were performed. Z-score normalization was applied to ensure the uniformity in pixel values across all images, creating a standardized input for the DL model. Additionally, spatial resampling was employed to achieve a consistent resolution of 1 × 1 × 1 mm, promoting uniformity in spatial dimensions throughout the dataset. These preprocessing steps were pipelined to ensure the reproducibility of this study. Finally, we generated 3D binary vessel masks by using a simplified U-Net architecture and the entire 3D binary vessel masks were fed into the DL model. The simplified network took CTA images as input, and the sigmoid activation function transformed the feature maps into probability maps. Subsequently, a threshold of 0.5 was usually applied to distinguish between vessel and non-vessel regions (Ma et al., 2021). Notably, the imaging data of DL development and the clinical experiment had equal scan and preprocessing protocols in order to avoid the introduction of bias.

### 2.3 CTA image annotations

Manual labeling of 42 arterial segments was used as the reference standard to develop the algorithm and evaluate the performance of the proposed model. Trained annotators labeled 42 arterial segments according to the anatomical segments of cerebral vessels (Bouthillier, van Loveren, and Keller, 1996; Yavagal and Haussen, 2011; Harrigan and Deveikis, 2018): including 7 segments of the ICA (C1-C7), 3 segments of the middle cerebral artery (MCA: M1, M2, M3-4), 4 segments of the anterior cerebral artery (ACA: A1, A2, A3, and A4-5), 3 segments of the posterior cerebral artery (PCA: P1, P2, and P3-4), 2 segments of the intracranial vertebral artery (VA: V3 and V4), as well as the posterior communicating artery (PCoA), anterior communicating artery (ACoA), and the basilar artery (BA).

Furthermore, the IAs were also manually labeled. The labeled results were confirmed by two specialized radiologists with 10, 11 years of experience. Disagreements of the two radiologists were arbitrated by a third specialized neuroradiologist. All the annotation was performed using software (3D Slicer Version 4.10.1; https://www.slicer.org).

### 2.4 Model training for automated labeling of the intracranial arteries

**FIGURE 2:**
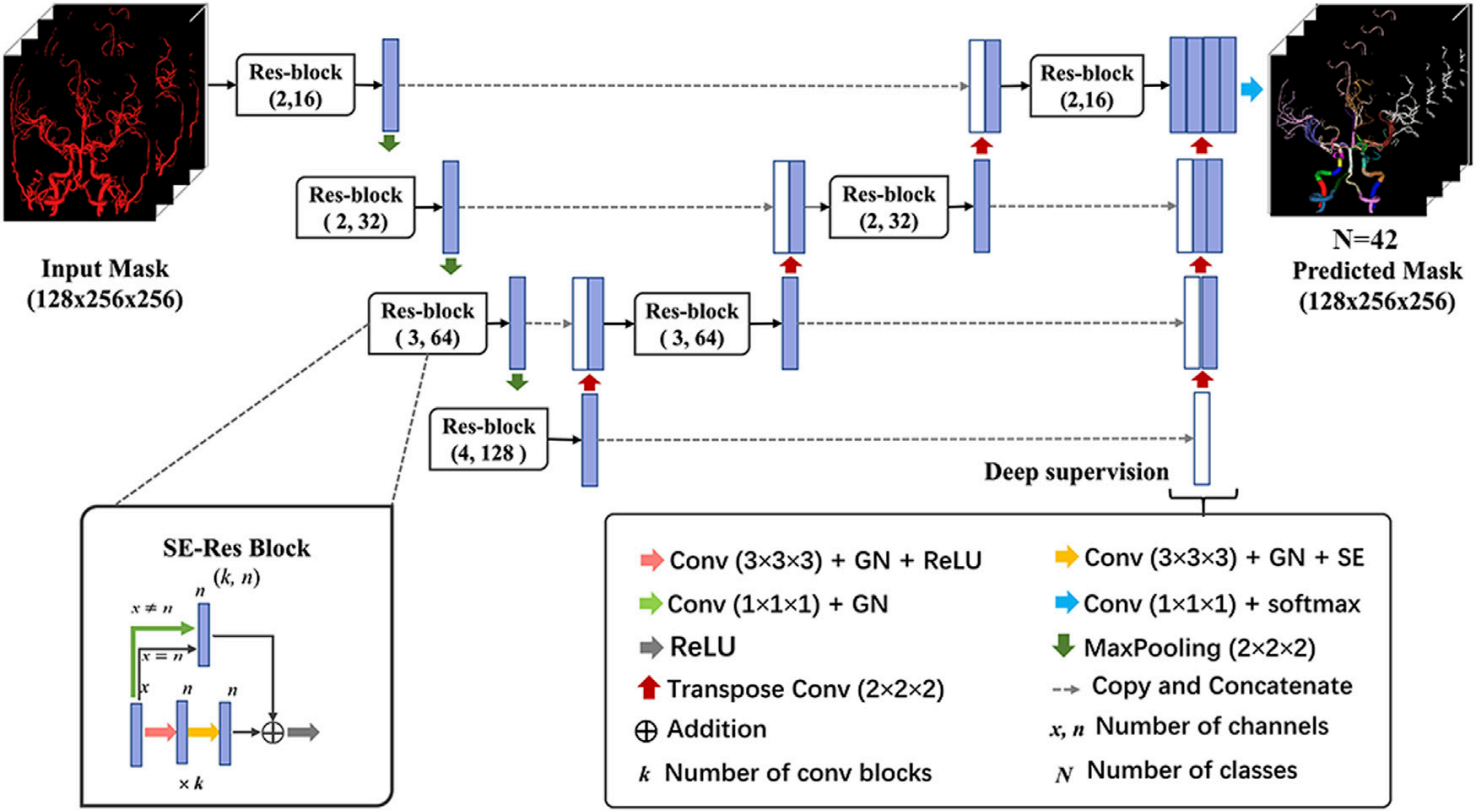
Architecture of the 3D network model for cerebral artery labeling. The proposed labeling model has an encoder-decoder architecture as popular U-Net, and the network takes in an input of preprocessed vessel masks and outputs the predicted probability of class for each voxel. The SE-Res block and deep supervision module are used to achieve better labeling performance of the network. 3D network, 3-dimensional network; GN, group normalization; ReLU, rectified linear units; SE-Res block, squeeze, and excitation-residual block.

A 3-dimensional convolutional neural network (CNN) was devised based on the U-Net architecture in this study (Figure 2). Specifically, the network comprised the encoding and decoding paths. Traditional convolution block was replaced by Squeeze-and-Excitation Residual (SE-Res) block, which consists of *k* cascading 3 × 3×3 convolutional layers of *n* channels involved with the group normalization (GN) and rectified linear units (ReLU) and *k* cascading 3 × 3×3 convolutional layers of *n* channels involved with the GN and SE. For better convergence, each block incorporated a skip connection with a 1 × 1×1 convolutional layer to reduce the number of channels. The encoder and decoder paths were augmented with the SE-Res blocks by means of channel-wise attention mechanisms, stable training as the depth of the network increased and adaptive information extraction of the feature map (Wang et al., 2021). In addition, the number of channels (*n*) was doubled after max pooling and was halved after transpose convolution. Furthermore, the deep supervision module was utilized in the decoder for faster convergence and better performance of the network via more direct learning process of the hidden layers (Lee et al., 2015). It included extra auxiliary branches at different stages of the decoder, allowing for the extraction of feature maps at various resolutions. The combination of SE-Res blocks and the deep supervision branch contributed to the model’s performance to capture both local and global contextual information. Ultimately, feature channels of the encoder were concatenated with the corresponding tensors of the decoder to merge advanced semantic information with low-level positional information.

During the training phase, CTA images with healthy vessels or stenosis after preprocessing were randomly cropped to 128 × 256×256 pixels and then fed into the vascular labeling model. The sliding window technique was applied to handle the volume during inference time. The network was trained using compound loss function with Dice loss and cross entropy, which is robust on highly imbalanced segmentation tasks. The Adam optimizer with a learning rate of 0.001 and decay rate of 0.98 was utilized to optimize the objective function. The batch size was set to 1 due to the limitation of memory and the number of training epochs was 300. Data augmentations such as random cropping, scaling, rotation, and elastic transformation were applied to CTA scans for learning inherent features and avoiding the overfitting problem. The training was implemented by using the Keras library of the Tensorflow backend on the workstation with a single V100 NVIDIA GPU.

### 2.5 Evaluation metrics of arterial labeling

Labeling performance is evaluated by determining three metrics: (1) Dice similarity coefficient (DSC), (2) Mean surface distance (MSD; [mm]) and (3) Hausdorff distance (HD; [mm]). The DSC ranges from 0 to 1, where value of 1 indicates high similarity. For MSD and HD, low values indicate high similarity. These three metrics are defined as follows (Hameeteman et al., 2011; Benkarim et al., 2021):

$$DSC\left(G,P\right)=\frac{2\left|G\cap P\right|}{\left|G\right|+\left|P\right|}=\frac{2\sum GP}{\sum G+\sum P}$$

Where G is the cerebral artery region in the ground truth and P is the predicted result.

$$MSD\left(G,P\right)=\frac{1}{\left|S\left(G\right)\right|+\left|S\left(P\right)\right|}\left(\sum_{p\in S\left(P\right)}\min_{g\in S\left(G\right)}∥p-g∥+\sum_{g\in S\left(G\right)}\min_{p\in S\left(P\right)}∥g-p∥\right)$$

Where S (.) denotes the set of surface voxels.

$$HD\left(G,P\right)=\max\left(\max_{g\in S\left(G\right)}\min_{p\in S\left(P\right)}∥g-p∥,\max_{p\in S\left(P\right)}\min_{g\in S\left(G\right)}∥p-g∥\right)$$

### 2.6 Clinical experiment

Besides the performance of model on voxel-wise segmentation, clinical utility of the model was validated in a real-world clinical scenario for IA localization. A total of 5 clinicians (W.Y., J.L., S.M.G., D.C.W., and J.J.) with different experience levels (5,3,3,1, and 1 year, respectively) participated as readers in the diagnostic accuracy study and identified the precise location of IA without and with algorithm assistance. The clinicians were blinded to clinical histories and read independently in a diagnostic reading room by software (3D Slicer). Following a washout period of 14 days, the examinations were interpreted again by the same corresponding clinician (if the first read was with aid of the algorithm, the second read was without algorithm assistance, and *vice versa*). Additionally, clinicians were provided with the model’s predictions in the form of 42 segments of vessels only when reading with algorithm assistance. Given the model prediction, readers took it into consideration or disregard it based on clinical judgment.

### 2.7 Statistical analysis

For comparison of labeling performance between the left and right vessels as well as the normal and stenotic vessels, the Wilcoxon signed rank test and Kruskal Wallis test were implemented, respectively. The proposed algorithm was assessed on CTA images with IAs by computing accuracy, interpretation time, and the interrater agreement of clinicians. The Wilcoxon signed rank test was used to assess differences in accuracy and average time of the clinicians with and without algorithm assistance. Furthermore, to investigate whether differences in their years of experience might impact the model’s usability and performance, five doctors with varying levels of experience were divided into two groups, i.e., high-level group of three doctors (5,3, and 3 years) and primary-level group of two doctors (both 1 year). The Kruskal Wallis test was implemented for comparison of the improved performance on identifying the precise location of IA between the high-level group and primary-level group. Considering over two readers and labels with no ranking or ordering, the interrater agreement of clinicians was determined using Fleiss’ Kappa (Fleiss and Cohen, 1973). To confirm whether model augmentation improved interrater agreement, the permutation test was performed on the difference between Fleiss’ Kappa of clinicians with and without model augmentation. The permutation procedure was repeated 10000 times to yield the null distribution of the Fleiss’ Kappa difference and the *p*-value was calculated as the proportion of the Fleiss’ Kappa differences that were higher than the observed Fleiss’ Kappa difference. A two-sided *p*-value less than 0.05 was considered statistically significant. Statistical analysis was conducted with IBM SPSS Statistics 23 (Armonk, New York) and Python 3.8 (Wilmington, Delaware).

## 3 Results

### 3.1 Patient and intracranial aneurysm characteristics

A total of 457 examinations (mean age, 51 years ±14 [standard deviation]; 209 female, 45.7%) and 96 examinations (mean age, 57 years ±10; 56 female, 58.3%) with 117 IAs (mean size, 5.1 mm ± 3.6) were used for the model development and the validation of its clinical utility. Table 1 shows the baseline characteristics of data set for labeling model development and Table 2 shows that of internal validation set for IAs localization.

**TABLE 1:** The baseline characteristics of data set for labeling model development.

Characteristics	Training set	Tuning set	Testing set	Total
No. of CTA	298	65	94	457
No. of Male	161	37	50	248
No. of Female	137	28	44	209
Age (mean ± SD)	51 ± 14	52 ± 13	51 ± 15	51 ± 14
Male	50 ± 13	54 ± 11	50 ± 17	51 ± 14
female	52 ± 15	50 ± 15	53 ± 12	52 ± 14
Vascular stenosis				
No	158	35	49	242
Mild (<50%)	76	14	19	109
Moderate (50%–70%)	13	4	7	24
Severe (>70%)	13	0	8	21
Occlusion (100%)	38	12	11	61

CTA, computed tomography angiography; SD, standard deviation.

**TABLE 2:** The baseline characteristics of internal validation set for IAs localization.

Characteristics	Internal validation set
No. of CTA	96
Sex	
No. of Male	42
No. of Female	54
Age (mean ± SD)	57 ± 11
Male	58 ± 11
female	57 ± 10
Multiplicity of IAs	117
No. of single IA	77
No. of multiple IAs	40
Size of IAs (mean ± SD, mm)	5.1 ± 3.6
No. of different IAs size	
<3 mm	26
3–5 mm	51
5–10 mm	31
>10 mm	9
No. of different IAs location	
ICA	69
MCA	24
ACA	4
ACoA	12
PCA	0
PCoA	1
BA	5
VA	2

ACA, anterior cerebral artery; ACoA, anterior communicating artery; BA, basilar artery; CTA, computed tomography angiography; IAs, intracranial aneurysms; ICA, internal carotid artery; MCA, middle cerebral artery; PCA, posterior cerebral artery; PCoA, posterior communicating artery; SD, standard deviation; VA, vertebral artery.

### 3.2 Labeling performance of the model

**FIGURE 3:**
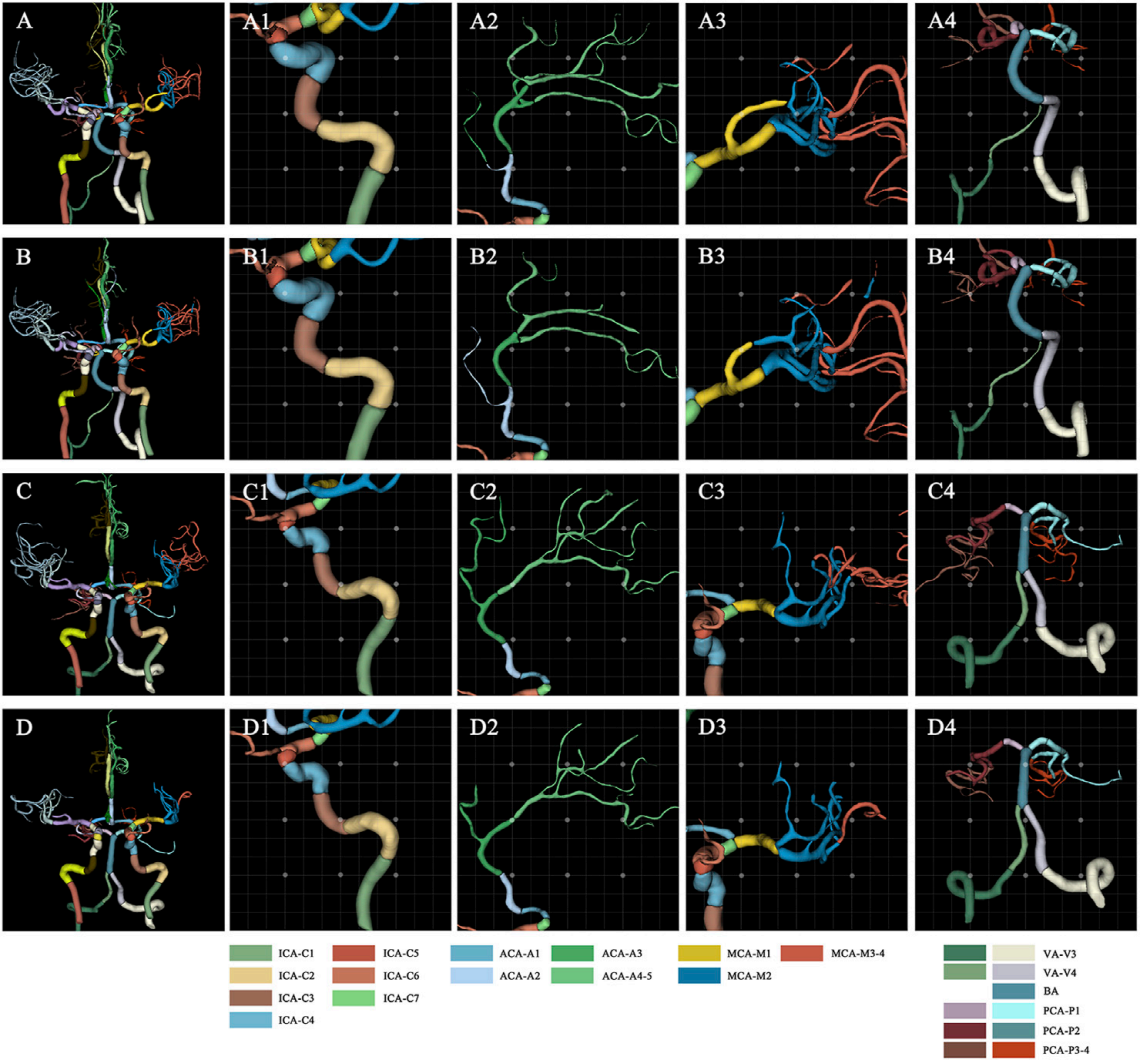
Visualization of manual and automated labeling for typical large vessels of two cases. For case 1 with a DSC of 0.85, MSD of 1.25, and HD of 10.71, the first raw provides the ground truth of detailed segments for the whole cerebral vessels **(A)**, ICA **(A1)**, ACA **(A2)**, MCA **(A3)** and VA with BA **(A4)**, which are displayed from left to right column. The second row represents the corresponding labeling results of the model, i.e., the whole cerebral vessels **(B)**, ICA **(B1)**, ACA **(B2)**, MCA **(B3)** and VA with BA **(B4)**. Similarly, for case 2 with a DSC of 0.88, MSD of 0.82, and HD of 5.91, the third raw provides the ground truth of detailed segments for the whole cerebral vessels **(C)**, ICA **(C1)**, ACA **(C2)**, MCA **(C3)** and VA with BA **(C4)**. The fourth row represents the corresponding labeling results of the model, i.e., the whole cerebral vessels **(D)**, ICA **(D1)**, ACA **(D2)**, MCA **(D3)** and VA with BA **(D4)**. ACA, anterior cerebral artery; BA, basilar artery; DSC, dice similarity coefficient; HD, Hausdorff distance; ICA, internal carotid artery; MCA, middle cerebral artery; MSD, mean surface distance; VA, vertebral artery.

The 3D network model for labeling of 42 arterial segments achieves promising performance in the testing set (94 cases) (Table 3). The 3D visualization of manual and CNN-automated labeling for 42 segments of vessels for two cases was shown in Figure 3. Overall, the model performs remarkably in labeling of 42 segments with the mean DSC of 0.88, MSD of 0.82 mm and HD of 6.59 mm. ICA consisting of 7 detailed segments obtained excellent results with DSCs ranging from 0.78 to 0.96, MSDs ranging from 0.24 mm to 0.60 mm, and HDs ranging from 1.67 mm to 4.70 mm Evaluation metrics show a decrease in labeling performance of MCA (M3-4), ACoA and PCoA. The evaluation metrics of large arteries were also calculated and described in Supplementary Table S1.

**TABLE 3:** Performance of the labeling model for 42 segments in test cohort.

Arterial segments	DSC ± std	MSD ± std (mm)	HD ± std (mm)
ICA			
C1	0.91 ± 0.12	0.24 ± 0.45	1.88 ± 3.33
C2	0.95 ± 0.04	0.57 ± 2.34	4.70 ± 16.83
C3	0.94 ± 0.05	0.47 ± 1.18	2.87 ± 5.37
C4	0.96 ± 0.05	0.19 ± 0.33	1.67 ± 1.66
C5	0.78 ± 0.17	0.60 ± 0.57	2.04 ± 1.22
C6	0.93 ± 0.07	0.51 ± 1.82	3.21 ± 7.98
C7	0.91 ± 0.08	0.35 ± 0.69	4.38 ± 13.24
MCA			
M1	0.94 ± 0.06	0.99 ± 2.60	6.01 ± 15.67
M2	0.88 ± 0.12	0.44 ± 0.80	3.74 ± 6.47
M3-4	0.56 ± 0.32	0.76 ± 2.46	11.37 ± 27.24
ACA			
A1	0.94 ± 0.12	0.15 ± 0.43	3.85 ± 13.00
A2	0.89 ± 0.14	1.81 ± 4.27	12.09 ± 18.77
A3	0.80 ± 0.23	2.31 ± 3.89	17.86 ± 16.92
A4-5	0.82 ± 0.20	1.60 ± 4.37	26.99 ± 18.7
PCA			
P1	0.89 ± 0.19	0.94 ± 5.15	4.02 ± 17.28
P2	0.95 ± 0.10	0.70 ± 4.84	4.42 ± 12.50
P3	0.86 ± 0.18	1.88 ± 6.57	8.82 ± 22.55
VA			
V3	0.96 ± 0.11	0.48 ± 1.75	4.57 ± 15.86
V4	0.93 ± 0.10	1.13 ± 3.54	6.02 ± 14.19
ACoA	0.66 ± 0.31	0.57 ± 0.63	2.28 ± 2.04
PCoA	0.76 ± 0.36	0.16 ± 0.34	1.72 ± 2.79
BA	0.97 ± 0.03	0.65 ± 2.08	3.49 ± 9.68
42 segments	0.88 ± 0.19	0.82 ± 3.10	6.59 ± 15.64

ACA, anterior cerebral artery; ACoA, anterior communicating artery; BA, basilar artery; DSC, dice similarity coefficient; HD, hausdorff distance; ICA, internal carotid artery; L, left; MCA, middle cerebral artery; MSD, mean surface distance; PCA, posterior cerebral artery; PCoA, posterior communicating artery; R, right; VA, vertebral artery.

**FIGURE 4:**
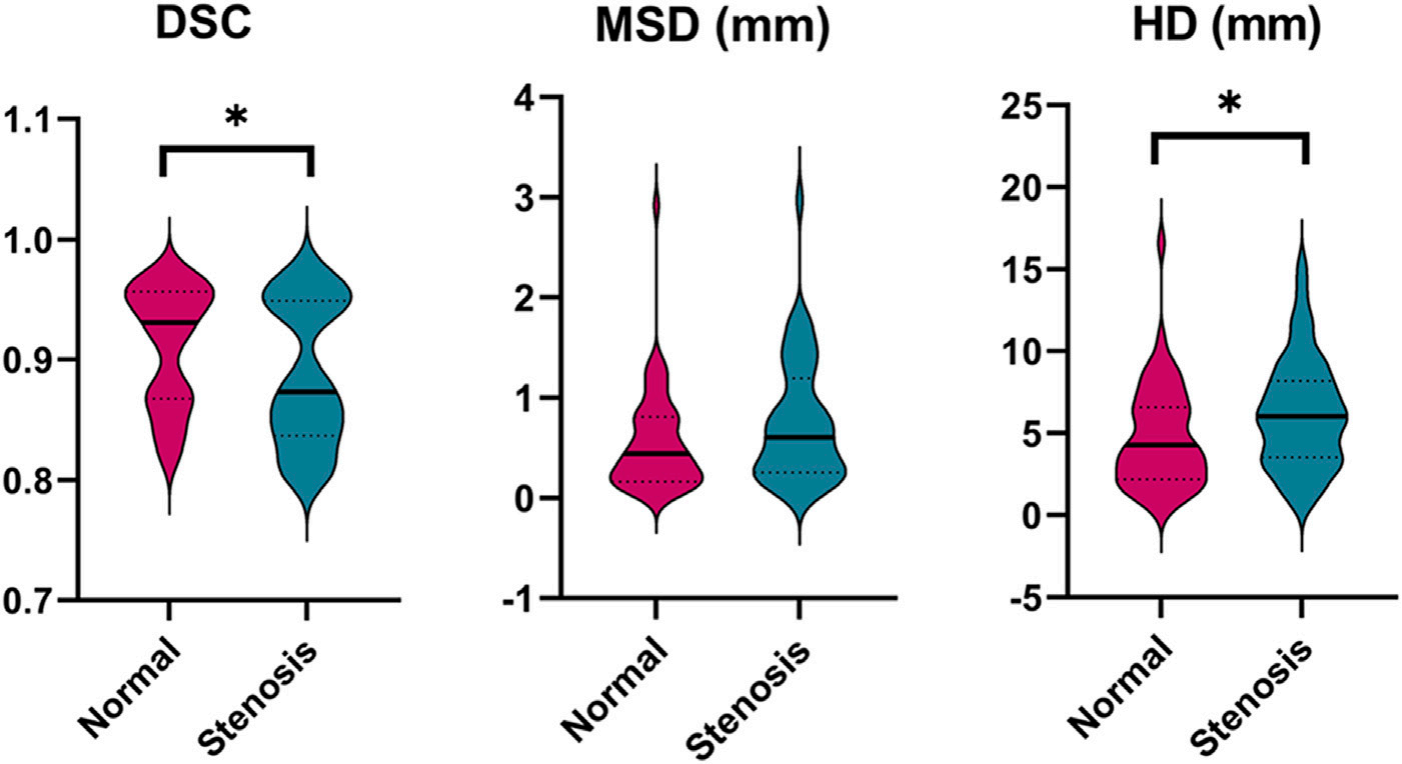
Comparison of the labeling performance on the normal and stenotic vessels. Violin plots are used to show the distribution of three metrics and visualize the statistic results. *p < 0.05; **p < 0.01. DSC, dice similarity coefficient; MSD, mean surface distance; HD, Hausdorff distance.

Besides, the statistical differences between the left and right labeling metrics of vessels were analyzed and the results are presented visually in Supplementary Figure S1 as a violin plot. Clearly, right ICA has a higher DSC value of 0.91 compared to that of 0.90 of the left (*p* < 0.001). It is noticeable that model performance of left PCoA outperforms that of the right in terms of MSD and HD corresponding to 0.09 mm vs. 0.10 mm (*p* = 0.005) and 1.15 mm vs.1.40 mm (*p* = 0.017). Regarding the differences of labeling performance on normal and stenotic vessels, healthy vessels have better labeling results as depicted in Figure 4, particularly for DSC (0.91 vs. 0.89; *p* = 0.047) and HD (4.75 mm vs. 6.19 mm; *p* = 0.028).

### 3.3 Clinical performance on precise IAs localization

**FIGURE 5:**
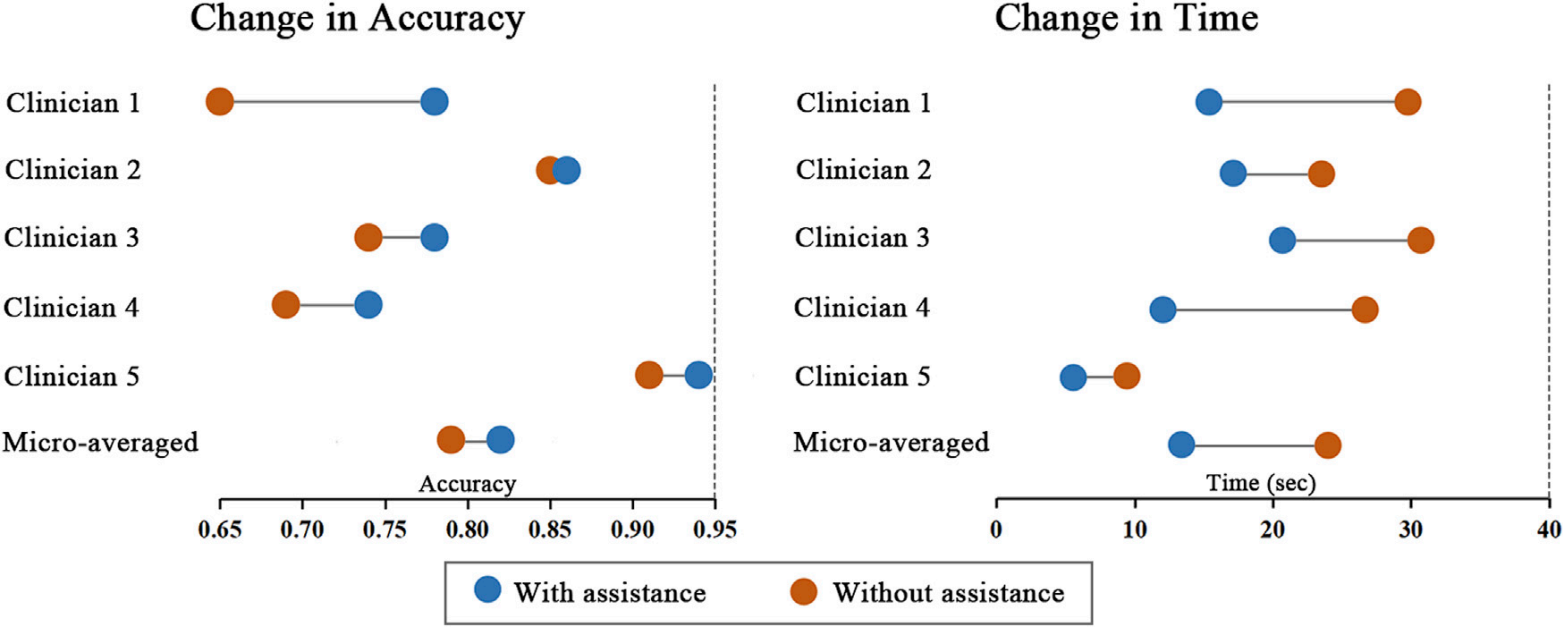
Change in individual clinicians’ performance metric. Horizontal lines depict the change in performance metric for each clinician with and without model assistance. The orange dot represents performance without model, and the blue dot represents performance with model assistance.

**FIGURE 6:**
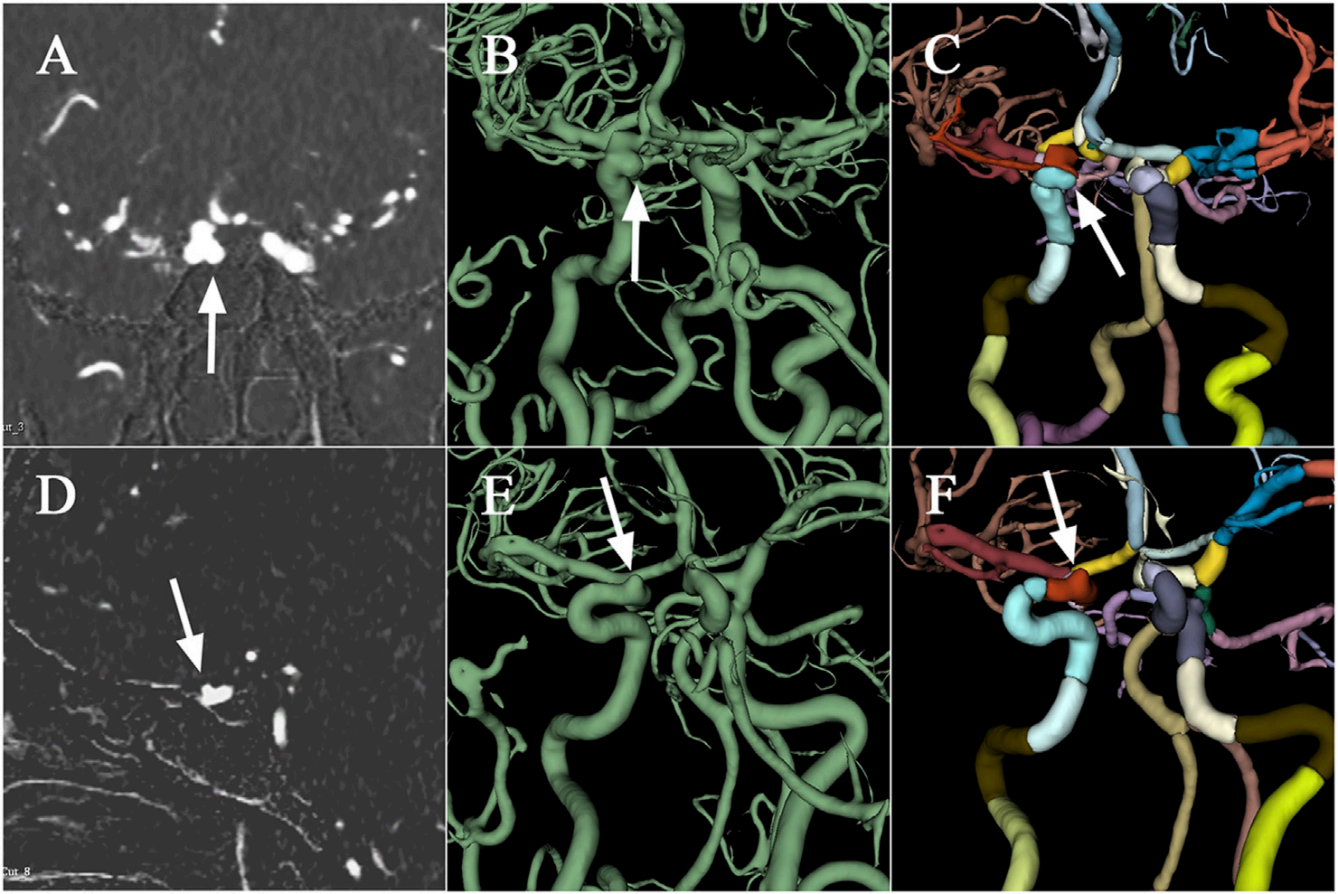
Examples of aneurysms in the validation dataset. The first raw presents CTA scan **(A)**, binary mask **(B)**, and the labelled arteries **(C)** with IA located on R-C5 of ICA, which are displayed from left to right column. Similarly, the second raw depicts CTA scan **(D)**, binary mask **(E)**, and the labelled arteries **(F)** with IA located on R-C6. CTA, computed tomography angiography; IA, intracranial aneurysm; ICA, internal carotid artery; R, right.

Performance improvements in terms of accuracy, interpretation time per case and interrater agreement across clinicians when determining the precise location of IA are reported in Table 4, and individual clinician improvement is detailed in Figure 5. More precisely, clinicians achieved a mean accuracy of 0.82 (95% confidence interval (CI), 0.79–0.86) with model augmentation and there was a statistically significant increase in the mean accuracy (0.04; 95% CI, 0.01 to 0.08; *p* = 0.003). Additionally, the mean time per case across clinicians was 14.14 s (95% CI, 10.03–18.25 s) with assistance and the time to diagnosis was significantly lower (difference, −9.76 s; 95% CI, −17.06 to −2.45 s; *p* < 0.001) compared to that of clinicians without assistance. For the clinicians, there was a significant increase of 0.07 (*p* = 0.029) in their interrater agreement, with a Fleiss’ Kappa of 0.59 without assistance and 0.66 with assistance. Individual performances with and without algorithm assistance were shown in Supplementary Table S2. Comparison of clinical performance of clinicians with primary-level and high-level experience when interpreting the precise location of IA was provided in Supplementary Table S3, which indicates that the accuracy is not affected by the doctor‘s experience but interpretation time of primary-level clinicians is enhanced better (difference, −14.39 s; 95% CI, −17.18 to −11.60; *p* < 0.001) compared to that of high-level clinicians. Figure 6 depicts two examples of aneurysms located on R-C5 and R-C6 in the validation dataset.

**TABLE 4:** Clinical performance with and without algorithm assistance to predict precise location of IAs in internal validation cohort.

Metric	Without assistance (95%CI)	With assistance (95%CI)	Mean difference (95%CI)	*p*-Value
Accuracy	0.79 (0.75–0.83)	0.83 (0.80–0.86)	0.04 (0.01–0.08)	0.003
Time (s/case)	23.90 (17.79–30.00)	14.14 (10.03–18.25)	−9.76 (−17.06 to −2.45)	<0.001

CI, confidence interval.

## 4 Discussion

In this study, we developed a DL model firstly based on the CTA scans to automatically label intracranial arteries with 42 anatomical segments with the largest dataset. The proposed model exhibited notable labeling performance with the mean DSC of 0.88, MSD of 0.82 mm and HD of 6.59 mm. Furthermore, the model demonstrated superior labeling performance in healthy subjects compared to patients with stenosis (DSC: 0.91 vs. 0.89, *p* < 0.05; HD: 4.75 vs. 6.19, *p* < 0.05). Additionally, a clinically relevant set for the localization of IA was used to assess the model’s clinical utility and results showed that clinicians with model predictions achieved significant improvements when interpreting the precise location of IA.

Previous studies involve mainly atlas construction-based matching of arterial branches (e.g., UBA167) and sophisticated graph-based function processing for labeling of major intracranial arteries on MRA images (e.g., ICA, MCA, and ACA) with overall accuracies ranging from 93% to 96% or F1 score around 0.85 (Dunas et al., 2016; Robben et al., 2016; Dunas et al., 2017). However, these researches neglect the detailed anatomical segments of arteries and suffer from the common limitations due to the relatively small datasets and the subjects without pathological variability, which may have ramifications on the robustness and the clinical utility of algorithm performance. A network has been recently adopted to obtain the exhaustive anatomical classification of the 62 cerebral branches with accuracies ranging from 73% to 100% and F1 scores ranging from 0.67 to 0.99, whereas quantified geometric vessel features are required in advance and only the healthy subjects are utilized for this model development (Hong et al., 2023). Besides, 24 classes of arterial segments have been distinguished via a multi-scale U-Net architecture with macro F1score of 0.89 and balanced class accuracy of 0.83 in labeling detailed segments. However, it considers only the large artery rather than detailed segments of ICA whereas stenosis and aneurysms frequently occur (Hilbert et al., 2022). In this paper, we leveraged voxel-wise segmentation indices instead of classification indices, i.e., DSC, MSD and HD, to evaluate the performance of labeling model, given the anatomical morphology and 3D properties of vessels. The proposed model achieved the overall DSC of 0.88, MSD of 0.82 mm and HD of 6.59 mm, demonstrating superior results compared with the conventional segmentation of carotid lumens and the whole cerebral vessels (Hemmati et al., 2017; Chen et al., 2021; Guo et al., 2021; Huang, Wang, and Li, 2023). Besides, we found that labeling performance of MCA, ACoA and PCoA seemed to decline in line with prior researches (Dunas et al., 2017; Hilbert et al., 2022; Hong et al., 2023). This may attribute to the small diameter of distal MCA (i.e., M3-4, See Table 3) and the potential inter-individual variation of vessels.

As reported in many experiments, the DSC metric cannot fully express the performance of vascular segmentation (Li et al., 2020). Because minimal changes may lead to low DSC in case of small volumes and the DSC would remain high regardless of critical errors in relatively large volumes. Consequently, we also took the spatial distance-based metrics (i.e., MSD and HD) to evaluate the surface coincidence and the segmentation quality of outliers, respectively. In Supplementary Figure S1, our result suggested that there were significant differences between left and right ICA (DSC: 0.90 vs. 0.91, *p* < 0.01) and PCoA (MSD: 0.09 vs. 0.10, *p* < 0.01; HD: 1.15 vs. 1.40, *p* < 0.05). In terms of DSC metric vulnerable to the variation of vascular shape, the significant difference of left and right ICA may be attributed to the asymmetrical nature *per se* (about 6%) and relatively high incidence of arterial stenosis compared to other arteries (Mujagic et al., 2016). For PCoA, the meta-analysis indicates the prevalence of PCoA hypoplasia or aplasia is almost up to 43%, which is likely to be the primary factor that leads to the difference of labeling performance in terms of distance-based metrics (Jones et al., 2021). Furthermore, we achieved a higher level of labeling performance in healthy controls compared with that of patients with stenosis (See Figure 4), providing the evidence that pathological variations of cerebral vasculature results in the lower prediction performance (DSC: 0.91 vs. 0.89, *p* < 0.05; HD: 4.75 vs. 6.19, *p* < 0.05). Patients with different level of stenosis often showed a lack of vascular volume, changes in vascular surface texture, and even partial cerebrovascular loss by means of observing CTA scans, which seems to account for our finding.

We designed a validation process to simulate the clinical scenario of precise IA localization since the location of aneurysm is critical for the growth and rupture risk, clinical decision, and outcome evaluation (Investigators et al., 2012; Thompson et al., 2015). In our study, with model augmentation, the mean accuracy, time to diagnosis and interrater agreement of aneurysm localization across clinicians significantly improved, suggesting that the proposed algorithm seems to assist clinicians with varying level of experience in higher efficiency of diagnosis, more accurate and more consistent clinical interpretations. Additionally, the proposed model has great potential in multiple clinical application aspects. It enables stenosis localization and the automatic quantification of specific segments of blood vessels such as arterial diameter, volume, cross-sectional area (narrowing grade), curvature index, even hemodynamic parameters, thus providing additional guidance for future research and treatment of cerebrovascular diseases.

There exist several limitations. First, this study was conducted on data from a single institution. Hence, the generalizability of the algorithm entails further assessment on multicentric external data and there are challenges in identifying precise location of other vascular lesion such as arteriovenous malformation. Second, the model’s labeling performance on vascular segments for small diameter (e.g., distal MCA) and high incidence variation (e.g., PCoA) was slightly weakened. The model may be matured if self-attention mechanism is incorporated by learning rich hierarchical representations of curvilinear structures (Mou et al., 2021). Also, since we focused on the cerebral vasculature in CTA images, the model’s performance on other imaging modalities remains unknown.

## 5 Conclusion

The precise anatomical labeling of intracranial arteries is a fundamental step in automated diagnosis and decision-making processes for various arterial diseases, and it remains challenging despite considerable research efforts. We developed a powerful DL model to automatically label 42 intracranial arteries segments on CTA images, demonstrating superiority over existing models. Additionally, a significant improvement in clinicians’ performance to precisely locate IAs was observed when assisted by proposed model. This research represents an initial stride towards a more comprehensive evaluation of labeling algorithms and underscores the immense potential of such advancements in the field of computer-aided medicine.

## Data Availability

The raw data supporting the conclusion of this article will be made available by the authors, without undue reservation.
