# Supplementary material for: Automated anatomical labeling of the intracranial arteries via deep learning in computed tomography angiography
Source: Front Physiol. 2024 Jan 4;14:1310357. doi: 10.3389/fphys.2023.1310357 (PMC10794642; doi:10.3389/fphys.2023.1310357)
Supplement: Supplementary file 1 [file Table1.DOCX]

Supplementary Material

# Supplementary Figures and Tables

## Supplementary Tables

**Supplementary Table 1.** The evaluation metrics of large arteries.

| Arteries | DSC ± std | MSD ± std(mm) | HD ± std(mm) |
| --- | --- | --- | --- |
| L_ICA | 0.90±0.11 | 0.36±0.85 | 2.93±7.82 |
| R_ICA | 0.91±0.11 | 0.48±1.60 | 3.00±10.17 |
| L_MCA | 0.79±0.26 | 0.90±2.40 | 8.02±18.09 |
| R_MCA | 0.80±0.26 | 0.56±1.81 | 6.05±19.37 |
| L_ACA | 0.87±0.17 | 1.48±3.85 | 16.15±19.16 |
| R_ACA | 0.85±0.21 | 1.47±3.60 | 14.33±18.77 |
| ACoA | 0.66±0.31 | 0.52±0.54 | 2.04±1.78 |
| L_PCA | 0.90±0.16 | 1.44±3.82 | 6.46±13.41 |
| R_PCA | 0.90±0.17 | 1.05±3.71 | 5.24±9.42 |
| L_PCoA | 0.75±0.37 | 0.09±0.20 | 1.15±1.29 |
| R_PCoA | 0.77±0.34 | 0.10±0.23 | 1.40±2.12 |
| BA | 0.97±0.03 | 0.65±2.07 | 3.49±9.68 |
| L_VA | 0.94±0.09 | 0.86±2.79 | 5.87±11.79 |
| R_VA | 0.94±0.12 | 1.04±3.26 | 5.16±11.26 |

ACA, anterior cerebral artery; ACoA, anterior communicating artery; BA, basilar artery; DSC, dice similarity coefficient; HD, Hausdorff distance; ICA, internal carotid artery; L, left; MCA, middle cerebral artery; MSD, mean surface distance; PCA, posterior cerebral artery; PCoA, posterior communicating artery; R, right; VA, vertebral artery.

**Supplementary Table 2.** Individual performances with and without algorithm assistance.

| Metric | Without assistance  (95%CI) | With assistance  (95%CI) | Mean difference  (95%CI) |
| --- | --- | --- | --- |
| Clinician 1 |  |  |  |
| accuracy | 0.91 (0.86 to 0.97) | 0.94 (0.90 to 0.98) | 0.03 (-0.02 to 0.07) |
| Time (s/case) | 9.30 (8.21 to 10.39) | 5.46 (4.85 to 6.08) | -3.84 (-4.76 to -2.92) |
| Clinician 2 |  |  |  |
| accuracy | 0.69 (0.61 to 0.78) | 0.74 (0.66 to 0.82) | 0.05 (-0.05 to 0.15) |
| Time (s/case) | 26.55 (22.70 to 30.39) | 11.94 (10.15 to 13.73) | -14.61 (-18.24 to -10.98) |
| Clinician 3 |  |  |  |
| accuracy | 0.74 (0.65 to 0.82) | 0.78 (0.70 to 0.85) | 0.04 (0.01 to 0.08) |
| Time (s/case) | 30.71 (0.67 to 60.74) | 20.75 (0.41 to 41.10) | -9.96 (-46.56 to 26.65) |
| Clinician 4 |  |  |  |
| accuracy | 0.85 (0.78 to 0.91) | 0.86 (0.80 to 0.93) | 0.02 (-0.07 to 0.11) |
| Time (s/case) | 23.40 (20.54 to 26.26) | 17.17 (14.45 to 20.20) | -6.23 (-9.02 to -3.43) |
| Clinician 5 |  |  |  |
| accuracy | 0.65 (0.56 to 0.74) | 0.78 (0.70 to 0.85) | 0.13 (0.03 to 0.22) |
| Time (s/case) | 29.61 (24.29 to 34.93) | 15.44 (13.01 to 17.87) | -14.17 (-18.47 to -9.87) |

CI, confidence interval.

**Supplementary Table 3.** Comparison of clinical performance of clinicians with primary-level and high-level experience when interpreting the precise location of IA.

| Metrics | Mean difference of two interpretations (95%CI) | | P value |
| --- | --- | --- | --- |
|  | Primary-level group | High-level group |  |
| Accuracy | 0.09 (0.02 to 0.16) | 0.03 (-0.01 to 0.06) | 0.066 |
| Time (s/case) | -14.39 (-17.18 to -11.60) | -6.66 (-18.72 to 5.39) | <0.001 |

CI, confidence interval; IA, intracranial aneurysm.

## Supplementary Figures


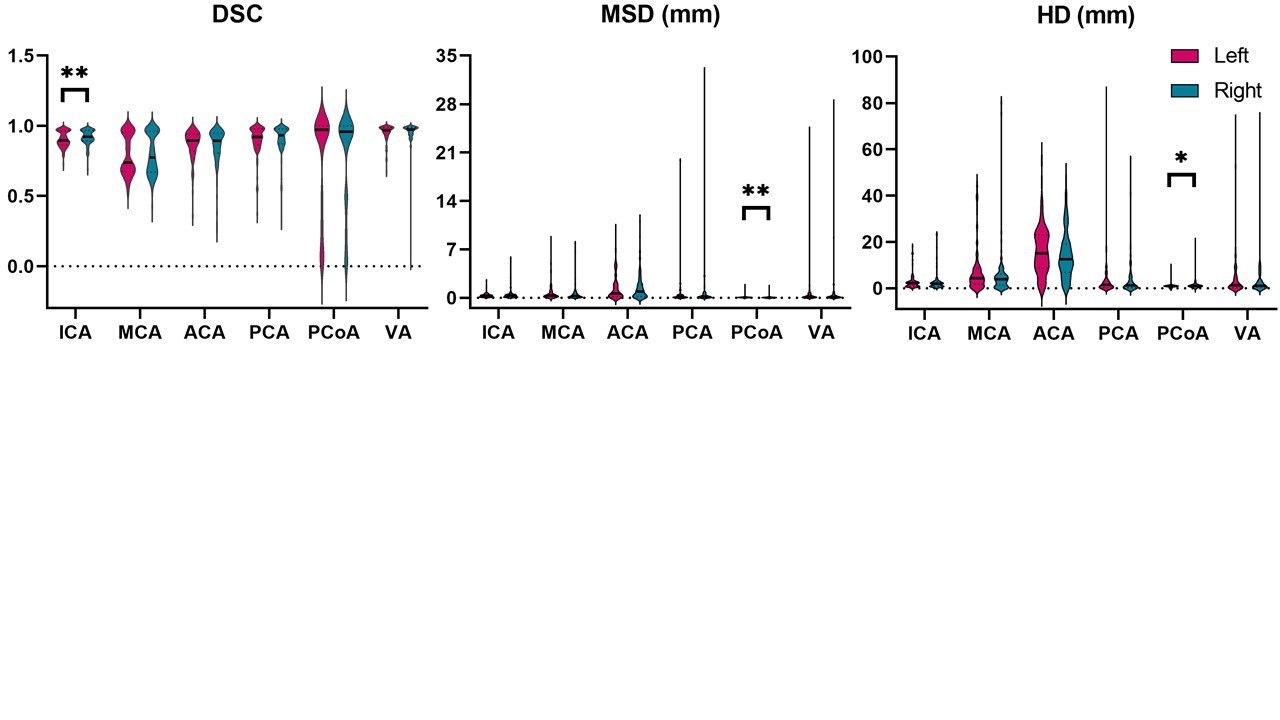


**Supplementary Figure 1.** Comparison of the labeling performance on the left and right vessels. Violin plots are used to show the distribution of three metrics and visualize the statistic results. *p<0.05; **p<0.01. ACA, anterior cerebral artery; DSC, dice similarity coefficient; HD, Hausdorff distance; ICA, internal carotid artery; MCA, middle cerebral artery; MSD, mean surface distance; PCA, posterior cerebral artery; PCoA, posterior communicating artery; VA, vertebral artery.
